# Good Quality Locally Procured Drugs Can Be as Effective as Internationally Quality Assured Drugs in Treating Multi-Drug Resistant Tuberculosis

**DOI:** 10.1371/journal.pone.0126099

**Published:** 2015-04-29

**Authors:** Ejaz Qadeer, Razia Fatima, Katherine Fielding, Fahad Qazi, David Moore, Mishal S. Khan

**Affiliations:** 1 National Tuberculosis Control Program Pakistan, Islamabad, Pakistan; 2 London School of Hygiene and Tropical Medicine, London, United Kingdom; 3 Research Alliance for Advocacy and Development, Karachi, Pakistan; Institut de Pharmacologie et de Biologie Structurale, FRANCE

## Abstract

**Background:**

Owing toGiven the high costs of drugs to treat multi-drug resistant tuberculosis (MDR-TB), the Green Light Committee (GLC) initiative enables TB programs to procure quality-assured drugs at reduced prices. Despite price reductions, internationally quality assured (IQA) drugs can be more expensive than locally procured drugs. There is little evidence to inform decision-makers about whether IQA drugs are more effective than local drugs. This is the first study to compare outcomes between MDR-TB patients treated using IQA, and locally procured drugs in the same hospitals during the same time period.

**Methods/Findings:**

A retrospective cohort study was conducted in three hospitals across Pakistan. Data on baseline characteristics and treatment outcomes during the first six months of treatment were extracted from hospital records of adult culture-positive pulmonary MDR-TB patients starting treatment between January 2011 and June 2012. Two cohorts were defined: patients receiving IQA drugs, and patients receiving locally procured non-IQA drugs. Data were analysed using Kaplan-Meier curves and Cox proportional hazards regression. The primary outcome compared between cohorts was time to culture conversion. Of 231 patients, 90 were in the IQA and 141 in the non-IQA cohorts. Baseline characteristics were similar except for higher frequency of quinolone resistance in the IQA cohort. Overall, 193 patients (84%) culture converted. Culture conversion was not faster in the IQA cohort; the median time was 81 and 68 days in the IQA and non-IQA cohorts, respectively. Unadjusted and adjusted hazard ratios for culture conversion in IQA verses non-IQA cohorts were 0.82 (95%-CI, 0.62-1.10) and 0.95 (95%-CI, 0.66-1.36) respectively.

**Conclusions:**

Use of good quality, locally procured drugs can be effective in treating MDR-TB, may involve lower costs than using IQA drugs and could strengthen developing country drug quality assurance systems. This may be a suitable alternative in lieu of or whilst awaiting arrival of internationally procured medicines.

## Introduction

Multi-drug resistant TB (MDR-TB), which is caused by strains of *Mycobacterium tuberculosis* resistant to both isoniazid and rifampicin, poses several challenges for National Tuberculosis Programs (NTPs). Expensive and sophisticated diagnostic methods are needed to confirm MDR-TB cases and effective treatment requires patients to take costly second-line anti-tuberculosis drugs for more than 18 months. Furthermore, the number of MDR-TB cases notified to NTPs in high burden countries is rising every year and is expected to keep growing. While 94,000 MDR-TB cases were diagnosed in 2012, reports suggest that less than 25% of the people who are estimated to have MDR-TB were identified[1].

A major barrier to provision of timely and effective treatment by NTPs is the high cost of quality-assured second line drugs (SLDs). In order to promote access to and rational use of SLDs for the treatment of MDR-TB, the WHO formed a multi-institutional partnership called the Green Light Committee (GLC)[2,3]. The GLC initiative enables approved MDR-TB programs to access high quality second-line drugs at reduced prices through the Global Drug Facility (GDF)[4]. The GDF has a quality assurance system in place to ensure that all drugs supplied to GLC-approved projects are safe, effective and appropriately labelled. Price reductions are achieved through negotiations with pharmaceutical companies and pooled procurement of drugs generating economies of scale. Despite the subsidies, procuring drugs internationally through the GDF can be more expensive and logistically challenging for already over-burdened NTPs than procuring drugs from the local market. It is estimated that the additional costs associated with international transport, customs clearance and further local distribution results in internationally quality assured (IQA) drugs being 15–20% more expensive than locally procured drugs. Concerns about use of locally procured drugs, on the other hand, relate to quality and effectiveness compared to IQA drugs. In Pakistan, pharmaceutical drugs are regulated under The Drugs Act 1976; licensed manufacturers of drugs are to comply with local Good Manufacturing Practices (GMP) guidelines which outline manufacturing, storage, quality control assays and documentation requirements. However there are limited resources to monitor drug quality once a manufacturer has been granted a licence, and variations in quality may occur after a drug has been licensed.

Evidence from existing literature on patient benefit through use of IQA drugs is limited. An analysis of the first five projects approved by the GLC, involving data from 1768 MDR-TB patients reported treatment success in 65%, default in 14%, failure in 7% and death rates of 11%[5]. In comparison, a meta-analysis of treatment outcomes from non-GLC approved programs, which included data from 21 countries reported similar treatment outcomes: 62% of patients had successful outcomes, while 13% defaulted, 8% failed therapy and 11% died[6]. A crude comparison therefore does not suggest better treatment outcomes among patients taking IQA drugs versus non-IQA drugs. To our knowledge, however, there are no studies which directly compare outcomes between patients on IQA and non-IQA approved treatment in the same settings during the same time period.

Pakistan ranks fifth in terms of global MDR-TB burden[1]. In 2010, the Pakistan NTP initiated a pilot program though which the country’s main MDR-TB hospitals were provided IQA drugs and social support, in the form of monthly basic food rations and transport reimbursement, for a fixed number of patients each year. IQA drugs for 235, 500 and 882 patients were obtained in the years 2010, 2011 and 2012 respectively. The drugs were distributed between the main public sector hospitals managing MDR-TB patients. When batches of IQA drugs were received by hospitals, confirmed MDR-TB patients waiting to initiate treatment and any cases diagnosed after drugs had been received were started on a course of IQA drugs. In some cases, hospitals would switch a patient who had recently started a course of non-IQA drugs to IQA drugs. When IQA drugs had run out at a hospital, newly diagnosed patients were treated using locally available drugs until IQA drugs became available again. There was no cost to the patient for treatment when either IQA or non-IQA drugs were used. For procurement of locally available drugs, large hospitals, such as the Ojha Institute of Chest Diseases (OICD) in Karachi, follow a system to ensure drug quality which involves selecting manufactures with good credentials, reviewing manufacturers’ literature on drug characteristics (bioavailability etc.), and conducting independent chemical analyses where possible.

The objective of this study was to compare response to treatment in patients treated using IQA drugs through the GLC initiative with those treated according to standard local protocols using non-IQA drugs in the same hospitals during the same time period. The question being addressed is whether IQA drugs are more effective in treating MDR-TB than locally procured drugs.

## Methods

### Setting and Participants

A retrospective cohort study was conducted in three hospitals in different regions of Pakistan: OICD (Karachi), Leprosy Hospital (Rawalpindi) and Institute of Chest Diseases Kotri (Hyderabad). Hospitals were included in the study if they were treating MDR-TB patients using both IQA and non-IQA drugs simultaneously. Hospitals that were treating patients with exclusively IQA or non-IQA drugs during the study period were not included as patient populations may differ.

Culture-positive adults who were enrolled on to treatment for pulmonary MDR-TB at the study hospitals between January 2011 and June 2012 were included in the study. Exclusion criteria were: age under 15 years, only extra-pulmonary TB diagnosed, culture positive at diagnosis but negative/missing culture result at treatment initiation and default immediately before initiating treatment. Patients who had received less than 30 days of non-IQA drugs before starting on IQA drugs for their entire treatment were included in the study and classified as receiving IQA drugs. Any patients who had been switched from non-IQA to IQA drugs more 30 days after treatment initiation were excluded from the study.

All patients received free individualized treatment regimens at the study hospitals. Sputum smears and cultures were performed at diagnosis, initiation of treatment and at monthly follow-up visits over the course of treatment. Patients were followed up for six months after treatment initiation.

### Definitions of outcomes and explanatory variables

Time to conversion of sputum mycobacterial cultures, which is recognized as the most important interim indicator of the efficacy of anti-TB treatment for MDR disease[7,8], was the primary outcome used to compare IQA and non-IQA approved treatment. Culture conversion was defined as two consecutive negative sputum cultures at least one month apart. Time to culture conversion was defined as the time between treatment initiation and the first of the two negative cultures. If culture conversion had not occurred, observations were censored at the earliest of death, default or at six months post treatment initiation.

Secondary outcomes were time to sputum smear conversion, weight gain six months after treatment initiation and occurrence of drug-related side effects during the first six months after treatment initiation. Time to sputum smear conversion was defined as the time between treatment initiation and the first of two negative sputum smear microscopy results. This analysis restricted to patients who had a positive sputum smear result at the time of treatment initiation. If a patient’s sputum remained smear-positive, observations were censored as previously described.

Among the explanatory variables, the initial drug regimen was defined as the number of drugs prescribed in the first month of treatment. Drugs that were removed from the regimen within 30 days of initiation were not counted. Drugs to which patient isolates is sensitive was defined as drugs given to the patient for at least one month to which there was no resistance detected during drug sensitivity testing. Period of treatment initiation was classified into three categories based on the calendar month during which a patient was started on treatment: January—June 2011, July – December 2011 and January 2012 – June 2012. Treatment initiation delay was defined as the time between a positive diagnostic culture for MDR-TB and treatment initiation. Previous first and second line TB treatments were defined as the most recent first and second line treatments received prior to MDR-TB treatment initiation at the study site, respectively. Initial sputum microscopy grading results were taken for the specimen submitted at the time of treatment initiation or within two weeks of treatment initiation if no sputum microscopy was conducted on the day of treatment initiation.

### Data sources

Data on patients’ baseline characteristics, TB treatment history, current disease severity and treatment progress indicators were extracted from hospital records for a period of six months following treatment initiation and directly entered into a database. Individual patient data were checked by a data supervisor on a weekly basis, and any data entry errors identified were corrected by referring to the hospital records. Two cohorts of patients were defined: those who received IQA drugs and associated social support through the GLC initiative (IQA cohort) and those who received locally procured drugs (non-IQA cohort).

### Study size

The sample size was determined by the number of MDR patients being diagnosed and treated using IQA or non-IQA drugs during the study period; a power calculation was conducted at the beginning of the study. Using NTP records it was estimated that 109 patients had initiated treatment on IQA drugs and

200 patients had initiated treatment on non-IQA drugs at the study hospitals between January 2011 and June 2012. It was expected that 20% of patients in the non-IQA cohort will culture convert by 3 months post treatment initiation, based on a previous study conducted in Pakistan[8]. We estimated that a programmatically important difference between the cohorts would be 15% (ie. 35% of patients in the IQA cohort culture convert by 3 months post treatment initiation). Based on these data we estimated the study will have a power of 82% with a two-sided type I error of 5%.

### Statistical analysis

Baseline characteristics of cohorts were compared using chi—square test for categorical variables and t-test for continuous variables. Cox proportional hazards regression was used to assess the effect of IQA versus non-IQA treatment on time to sputum smear and culture conversion. Unadjusted and adjusted hazard ratios of sputum smear and culture conversion in IQA and non-IQA cohorts were calculated. Potential confounders identified *a priori* based on literature were used in the adjusted model. The proportionality assumption of the Cox model was assessed graphically. Kaplan-Meier curves were used to show time to sputum smear and culture conversion by IQA and non-IQA cohorts. Weight gain between cohorts was compared using multiple linear regression to control for potential confounders. Frequency of reported side-effects was compared using the z-test. All analyses were conducted using Stata/ IC 11.0 (Stata Corp, College Station, TX, USA).

### Ethics Statement

This study received ethical approval from the Pakistan Medical Research Council and the London School of Hygiene and Tropical Medicine. Since pre-existing secondary patient data was analysed and there was no contact with patients during data collection, the institutional review boards did not require informed consent to be taken. All data abstracted from existing patient records were anonymised.

## Results

Of 314 patients listed in the MDR treatment registers at the study hospitals during the study period, 83 were excluded because they were either not resistant to both isonaizid and rifampicin, were culture-positive at diagnosis but culture-negative by the time of treatment initiation or defaulted immediately before initiating treatment. Thus 231 patients were included in the study and of these 90 were in the IQA cohort and 141 were in the non-IQA cohort.

Patient baseline characteristics were generally similar across cohorts as was TB treatment history (Table 1). The median age was 32 and 31 years and median weight was 42 and 40 kilograms in the IQA and non-IQA cohorts, respectively. Female patients comprised 41% and 48% of the IQA and non-IQA cohorts respectively. Overall, 58% of patients were resistant to five first-line drugs and 5% were resistant to injectable second line drugs. Resistance to quinolones however was more frequent in the IQA cohort (60% vs 43%). There were no notable differences between cohorts in the overall number of drugs or regimens prescribed apart from the choice of aminoglycoside antibiotic used; Kanamycin was used more frequently in the non-IQA cohort (70% in non-IQA cohort vs 11% in IQA cohort, p<0.01) and Amikacin was used more frequently in the IQA cohort (28% in non-IQA cohort vs 79% in IQA cohort, p<0.01). The treatment initiation delay of 54 days was similar in the two cohorts and the mean number of drugs in the initial regimen to which patient isolates were susceptible was 4.5 in the IQA cohort and 4.6 in the non-IQA cohort.

**Table 1:** Patient characteristics at baseline by cohort (n = 231).

	IQA drugs (n = 90)	Non-IQA drugs (n = 141)	p- value
Female sex; n (%)	37(41)	67(48)	0.34
Age in years; Mean (Range)	32(16–65)	31(16–70)	0.39
Weight in kg at treatment initiation; Median(IQR)	42(35–48)	40(36–46)	0.81
Hospital at which patients received MDR-TB treatment; n(%)			
* OICD (Karachi)*	66(73)	103(73)	<0.01
* ICDK (Hyderabad)*	15(17)	37(26)	
* LHR (Rawalpindi)*	9(10)	1(1)	
Previous first-line treatment; n(%)			
* None*	0(0)	1(0.7)	0.72
* Category I*	26(29)	35(25)	
* Category II*	64(71)	105(75)	
Previous second-line treatment; n(%)			
* Received*	8(9)	9(6)	0.61
* Not Received*	82(91)	132(94)	
Initial sputum microscopy result*; n(%)			
* Positive*	84(93)	133(94)	0.41
* Negative*	1(1)	4(3)	
Number of first line drugs to which patient is resistant additional to isoniazid and rifampicin; n(%)			
* No drugs*	0(0)	7(5)	0.13
* 1 drug*	12(13)	13(9)	
* 2 drugs*	26(29)	40(28)	
* 3 drugs*	52(58)	81(57)	
Resistance to injectable drugs	4(4)	8(6)	0.68
Resistance to quinolones	54(60)	61(43)	0.01
Resistance to any of PAS/Eto/Cs	10(11)	14(10)	0.77
XDR-TB patients	4(4)	4(3)	0.52
Treatment initiation delay in days; Median(IQR)	54(10–66)	54(4–75)	0.28
Initial drug regimen			
* Drugs received for at least one month; Mean(range)*	5.7(4–8)	5.7(3–7)	0.94
* Drugs received for at least one month to which the patient isolate is sensitive; Mean(range)*	4.5(0–6)	4.6(3–6)	0.14

IQA = Internationally quality assured; IQR = interquartile range; *PAS = Para aminosalicylic acid / Eto = Ethionamide / Cs = Cycloserine*

*9 patients had no sputum smear result within 2 weeks of treatment initiation

**Fig 1:**
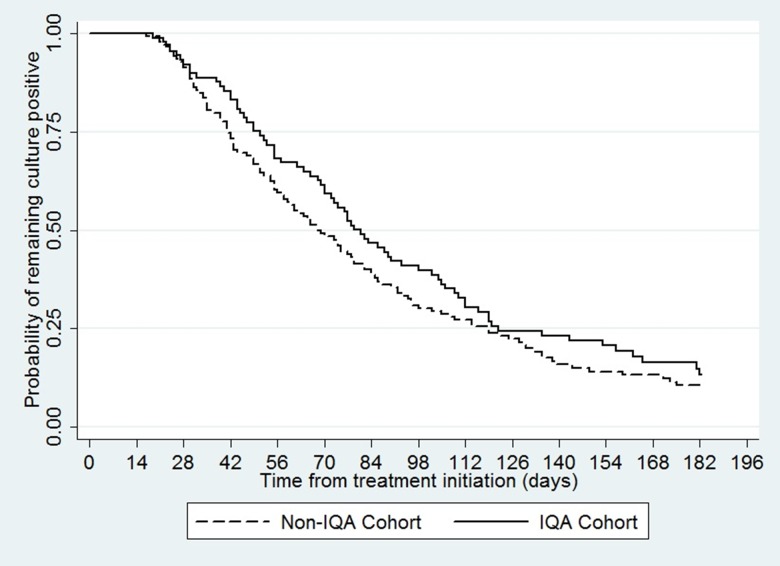
Kaplan-Meier curve showing time to culture conversion by cohort (n = 231).

One hundred and ninety-three patients (84%) culture converted within six months of treatment initiation. Median time to culture conversion was 81 days in the IQA cohort and 68 days in the non-IQA cohort (Table 2 and Fig 1). The unadjusted hazard ratio for culture conversion in the IQA verses non-IQA cohort was 0.82 (95% CI, 0.62–1.10; p = 0.19). After adjusting for potential confounding factors (age, gender, initial weight, previous first or second line drug use, sputum microscopy result at treatment initiation, resistance to first and second line drugs, number of drugs received to which the patient isolate was sensitive, period of treatment initiation, hospital at which patients received MDR-TB treatment and treatment initiation delay), the hazard ratio was 0.95 (95% CI, 0.66–1.36; p = 0.78).

**Table 2:** Treatment success indicators by cohort (n = 231).

	IQA drugs (n = 90)	Non-IQA drugs (n = 141)	p-value
Patients experiencing culture conversion; n(%)	74(82)	119(84)	0.66
Time to culture conversion in days; Median (IQR)	81(51–122)	68(41–119)	0.18
Cumulative probability of culture conversion at:			
* 60 days; % (95% CI)*	33(24–44)	44(36–52)	-
* 90 days; % (95% CI)*	58(48–68)	65(57–73)	-
* 180 days; % (95% CI)*	84(75–91)	90(84–94)	-
Patients experiencing sputum-smear conversion*; n (%)	71(85)	81(61)	<0.01
Time to sputum-smear conversion in days; Median (IQR)	70(44–121)	132(60–182.5**)	<0.01
Cumulative probability of sputum smear conversion at:			
* 60 days; % (95% CI)*	45(35–56)	25(19–34)	-
* 90 days; % (95% CI)*	65(54–75)	35(27–44)	-
* 180 days; % (95% CI)*	88(80–94)	65(56–73)	-
Weight at 180 days in kilograms; Median(IQR)†	44 (37–50)	45 (39–51)	-
Patients experiencing any side-effects; n(%)	34(38)	42(30)	0.21
Patients that defaulted or died within 6 months of initiating treatment; n(%)	11(12)	16(11)	0.84

CI = Confidence Interval, IQA = Internationally quality assured, IQR = Interquartile range

*Expressed as a percentage of patients who were smear-positive at treatment initiation (84 in the GLC group and 133 in the non-GLC group)

** Observations censored after 6 months (182.5 days); 66% converted by 6 months

† Data on weight gain at 6 months post treatment initiation available for 188 patients

**Fig 2:**
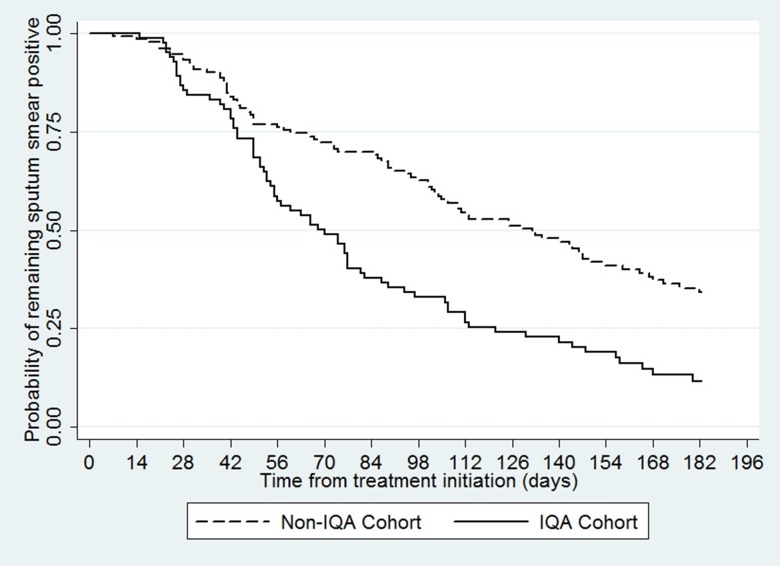
Kaplan-Meier curve showing time to smear conversion by cohort (n = 231).

Of the 231 culture-positive patients included in the study, 217 (94%) were also smear-positive and were included in the time to smear conversion analysis. Overall, 152 patients (70%) smear converted during the study period. Eighty-five percent (71/84) of patients in the IQA cohort and 61% (81/133) patients in the non-IQA cohort smear-converted within six months of treatment initiation. Unlike time to culture conversion, time to smear conversion was shorter in the IQA cohort (Median 70 days vs 132 days, Fig 2; adjusted hazard ratio, 2.34 [95% CI 1.58 – 3.46, p<0.01]).

To further understand the differences observed between cohorts in time to smear conversion, exploratory analyses were conducted. Comparing smear and culture results from the same specimen we found that in the IQA cohort 21% (19/90) of patients had culture positive and smear negative result compared with 49% (69/141) in the non-IQA cohort. Overall 74% of the discordant results had a scanty positive smear-microscopy grading (52% and 80% in the IQA and non-IQA cohorts, respectively). Culture-negative, smear-positive results were not found to cluster at a particular hospital or time period.

Data on weight at six months post treatment initiation was available for 84% (76/90) and 79% (112/141) of patients in the IQA and non-IQA cohorts. Median weight after six months of treatment was 44 kg and 45 kg in the IQA and non-IQA cohorts respectively. Weight gain in the IQA cohort was lower than that in non-IQA cohort even after adjustment for same factors described previously (adjusted difference in weight was 1.97 kg, 95% CI 0.47 kg to 3.48 kg; p = 0.01). The proportion of patients who experienced drug-related side-effects was 38% and 30% (p = 0.21) in the IQA and non-IQA cohorts respectively.

## Discussion

Results of this study indicate that there was very little difference in the primary outcome, time to culture conversion, between IQA and non-IQA cohorts. Good culture conversion rates were achieved using both types of drugs; more than 75% of patients treated using IQA or non-IQA drugs had sputum cultures negative for mycobacterial growth by the time they had completed four months of treatment. Median times to culture conversion in this study were substantially shorter than the 196 days previously reported in a single-centre study in Pakistan for patients on non-IQA drugs[8]. However, the median time to culture conversion in our study was in line with data previously reported from Latvia[7], Tanzania[9] and China[10].

An analysis of other treatment success indicators showed that patients in the IQA cohort did not experience greater weight gain (if anything slightly lesser weight gain) despite provision of food baskets to patients through the GLC program. Provision of food baskets may have an impact on adherence or weight gain later in treatment (ie. one year post treatment initiation); further studies to evaluate the strategy of providing nutritional support are required to draw conclusions. The frequency of self-reported side-effects did not differ between cohorts (p = 0.2). These clinical data are consistent with the culture outcome data of similar responses by IQA and non-IQA cohorts.

There was, however, strong evidence of faster time to smear-conversion in the IQA cohort; on average, patients on IQA drugs became negative on smear-microscopy almost two months before patients on non-IQA drugs. The same laboratories processed all samples at each site, laboratory technicians were unaware of whether patients were receiving IQA or non-IQA drugs, and there was no evidence of issues with the quality of laboratory results. The only difference in drug regimens between cohorts was in the choice of aminoglycoside antibiotic used (Kanamycin versus Amikacin), which should not cause differences in smear-conversion. Since the majority of smear-positive, culture-negative specimens were scanty positive, it is possible that non-viable bacteria may have been detected on these specimens. The presence of smear-positive, culture-negative results has been reported in other studies[11,12], but it is not clear why this phenomenon was more frequent in non-IQA cohort in the study. The apparent disconnect between smear and culture conversion between the two patient cohorts remains unexplained and warrants further investigation in other settings.

The challenge of diagnosing and managing increasing numbers of MDR TB patients is forcing NTPs in high burden countries to make difficult choices on resource allocation and program planning. With the wide-spread introduction of new diagnostics such as the Xpert MTB/RIF system, it is expected that there will be increasing numbers of newly diagnosed MDR-patients waiting to be put on treatment in high burden countries, and many countries are now reporting such “waiting lists”. A major decision that programs need to make is whether to wait to receive IQA drugs or to use lower cost drugs that are available locally. Until now evidence on whether there is a benefit to patients from being treated though IQA drugs has been lacking. This study is the first to directly compare response to treatment in IQA and non-IQA patient cohorts undergoing treatment at the same hospitals during the same time period, and to find no evidence of a faster time to culture conversion in patients treated using IQA drugs.

It must be considered that this was a non-randomized study and that the distribution of unidentified confounding factors may have been different across cohorts. Apart from higher fluoroquinolone resistance in GLC patients, which was adjusted for when calculating adjusted hazards ratios, there was no major difference across cohorts in the baseline variables we studied. However, there may be other confounders on which we did not collect data. As our follow-up period ended 6 months from the start of treatment, we were able to compare culture conversion between cohorts but were not able to assess overall treatment success rates and recurrence rates. We believe that evidence from this first cohort study comparing IQA and non-IQA drugs warrants a study with a longer follow-up period and, based on the evidence generated, an assessment of the costs and benefits of using internationally procured drugs for MDR-TB treatment.

An important factor to consider when interpreting our findings is that the quality of local drugs used in study hospitals was checked according to hospitals’ own guidelines. We recognize that ‘locally procured drugs’ encompasses a range of products which can include counterfeit and substandard drugs[13], and that the drugs used in study hospitals may not be representative of all second-line antibiotics available on the local market in Pakistan or in other countries. Nevertheless, results from this study indicate that use of good quality, locally procured drugs results in a similar time to culture conversion as use of IQA drugs.

In order to ensure that patients are receiving good quality drugs, NTPs and large hospitals may need to invest in drug quality assurance systems if drugs are being procured locally. The costs and workload involved in quality assurance would need to be compared with that of international procurement and local distribution. An advantage of local procurement and quality testing would be to encourage growth of the local pharmaceutical industry and to exert pressure on manufacturers to abide by quality standards. Furthermore, if there is sufficient evidence that good quality local drugs are no less effective than internationally procured drugs, it may be unethical to delay initiation of treatment until international drugs are available in country.
